# Postbiotics from *Lactobacillus delbrueckii* Alleviate Intestinal Inflammation by Promoting the Expansion of Intestinal Stem Cells in *S*. Typhimurium-Induced Mice

**DOI:** 10.3390/foods13060874

**Published:** 2024-03-14

**Authors:** Mengting Wang, Yuting Ren, Xin Guo, Yanxin Ye, Haining Zhu, Jiaqi Zhang, Zan Huang, Kaifan Yu

**Affiliations:** 1 Laboratory of Gastrointestinal Microbiology, Jiangsu Key Laboratory of Gastrointestinal Nutrition and Animal Health, College of Animal Science and Technology, Nanjing Agricultural University, Nanjing 210095, China; 2 National Center for International Research on Animal Gut Nutrition, Nanjing Agricultural University, Nanjing 210095, China

**Keywords:** postbiotics, *L. delbrueckii*, *S.* Typhimurium, intestinal inflammation, intestinal stem cells

## Abstract

Previous studies have demonstrated that *L. delbrueckii* plays beneficial roles in modulating the gut microbiota, enhancing the intestinal barrier, and promoting animal growth. Postbiotics have a similar or even superior effect in protecting intestinal health compared to probiotics due to their excellent stability, extended shelf life, and safety. However, the protective effects and underlying mechanism of postbiotics from *L. delbrueckii* in intestinal inflammation remain unclear. In this study, we demonstrated the beneficial impact of postbiotics from *L. delbrueckii* on intestinal health by establishing a *S.* Typhimurium-induced intestinal inflammation model in mice, which included inactivated bacteria and supernatant. The results revealed that the probiotics and postbiotics from *L. delbrueckii* increased the survival rate and body weight of *S*. Typhimurium-induced mice, increased the level of IL-10, and decreased the levels of TNF-α and IL-6, thereby alleviating intestinal inflammation. Meanwhile, treatment with postbiotics decreased the levels of D-LA, DAO, and LPS and promoted the expression of Occludin, ZO-1, and Claudin-1 in the serum and jejunum, suggesting an improvement in intestinal barrier function by postbiotics. Additionally, the postbiotics modulated gut microbial diversity, increased the ratio of *Firmicutes* and *Bacteroidetes*, and restored the abundance of *Muribaculaceae*, *Lachnospiraceae_NK4a136_groups*, and *Alloprevotella* in *S*. Typhimurium-infected mice. Moreover, postbiotics from *L. delbrueckii* promoted the expansion of intestinal stem cells (ISCs) and increased the numbers of Paneth and Goblet cells. Taken together, these data revealed the beneficial role of postbiotics from *L. delbrueckii* in protecting against intestinal inflammation by promoting the expansion of ISCs.

## 1. Introduction

Invasion by pathogens results in significant damage and inflammation in the intestine. *Salmonella* is one of the most important causes of foodborne illness that live in the gastrointestinal tract of humans and animals and remains a significant global contributor to disease and death [1]. *Salmonella*-induced mice are frequently used as models to study treatments for intestinal inflammation [2,3]. Probiotics are considered a novel alternative to antibiotics for treating pathogenic bacterial infections. They are valued for their ability to modulate the structure of the intestinal microbiota, promote intestinal epithelial regeneration, repair damage to the intestinal mucosa, and enhance host immune function [4,5,6]. However, the widespread application of probiotics is restricted due to issues such as limited safety, instability, and storage challenges [7]. Recent studies have highlighted the potential benefits of using inactivated microbial cells and probiotic metabolites to prevent intestinal diseases and promote gastrointestinal health in animals, garnering significant interest. Heat-killed *Bifidobacterium bifidum* B1628 demonstrated significant improvement in the inflammatory state and intestinal damage by modulating the intestinal microbiota in mice with DSS-induced colitis. [8]. The surface proteins of three *Lactobacillus* ameliorated colon damage by decreasing the activity of peroxidase and the level of TNF-α in mice induced by *E.* coli [9]. In 2021, the International Scientific Association for Probiotics and Prebiotics (ISAPP) defined “preparations of inanimate microorganisms and/or their components that are beneficial to the health of the host” as postbiotics [10].

*L. delbrueckii* is commonly utilized as a probiotic due to its unique physiological functions. Including 0.1% *L. delbrueckii* in the diet improves the structure of intestinal morphology, regulates the composition of cecum microbiota, and promotes growth in piglets [11]. *L. delbrueckii* TUA4408L and its extracellular polysaccharides have been found to have beneficial effects in activating the intestinal epithelial inflammatory response and preventing intestinal diseases by regulating Toll-like receptors 2 and 4 in *E. coli*-induced pigs [12]. Oral administration of *L. dellbrueckii* PTCC1057 reduced glucose levels and elevated Sestrin-3 levels in diabetic mice [13]. As a feed additive, *L. delbrueckii* has been shown to modulate intestinal microbiota, improve growth performance, increase the feed conversion ratio, and enhance immunity in pigs [14,15]. However, The role of the inactivated *L. delbrueckii* and metabolites of *L. delbruecki* in intestinal health remains to be clarified.

In this study, we aimed to investigate the effects of postbiotics from *L. delbrueckii* on intestinal inflammation in a mouse model induced by *S.* Typhimurium, which would provide support for postbiotics as a potential treatment for human intestinal diseases.

## 2. Materials and Methods

### 2.1. Bacterial Culture and Preparation of Postbiotics

*Lactobacillus delbrueckii* subsp. jakobsenii was isolated from the intestines of healthy piglets, and it is a subspecies of *Lactobacillus delbrueckii*. *Lactobacillus delbrueckii* subsp. jakobsenii (NCBI: txid1217420) belongs to the *Lactobacillus* genus of *Firmicutes*. We inoculated the activated bacteria in MRS medium and incubated them under anaerobic conditions at 37 °C and 100 rpm for 10 h until the later stage of logarithmic growth. To obtain *L. de.* p, the bacterial solution was centrifuged, and the precipitation was washed twice with sterile PBS. Then, we resuspended the bacteria to a concentration of 5 × 10^8^ CFU/mL. In order to obtain *L. de.* i, the resuspended bacteria were incubated in water at 100 °C for 20 min and then plated for 24 h to ensure the inactivation of the bacteria. The bacterial supernatant was filtered using a 0.22 μm water system filter to collect the *L. de.* s. *S*. Typhimurium (ST) was cultured in LB medium for 8 h. Subsequently, it was centrifuged and washed twice with sterile PBS, and the concentration was adjusted to 1 × 10^7^ CFU/mL.

### 2.2. Animals and Experimental Design

Five-week-old male mice (C57BL/6J, Jiangsu Jicui Yaokang Biotechnology Co., Ltd., Nanjing, China) were acclimated to SPF for one week and randomly divided into five groups for treatment. During the experiment, the mice were freely fed conventional feed and water. All experimental procedures were approved by the Laboratory Animal Welfare and Ethics Committee of Nanjing Agricultural University (NJAU No. 20221202229, approved on 25 November 2022).

The experimental design was as follows. These groups included (1) the normal group, where sterile PBS was administered orally for 19 days; (2) the ST group, where the mice were treated with sterile PBS orally for 0–14 days, treated with ST on the 15th day, and administered sterile PBS orally for 16–19 days; (3) the *L. de.* p + ST group, where mice were treated with *L. de.* p orally for 0–14 days, treated with ST on the 15th day, and treated with *L. de.* p orally for 16–19 days; (4) the *L. de.* i + ST group, where mice were treated with *L. de.* i orally for 0–14 days, treated with ST on the 15th day, and treated with *L. de.* i orally for 16–19 days; and (5) the *L. de*. s + ST group, where mice were treated with *L. de.* s orally for 0–14 days, treated with ST on the 15th day, and treated with *L. de.* s orally for 16–19 days. There were 15 mice in each group. To heighten the intestinal susceptibility to *ST*, the mice were deprived of food and water for 4 h before and after being treated with *ST*. Each mouse was orally administered *ST* at a dose of 1 × 10^7^ CFU. After 24 h of treatment, feces from mice were collected and coated on solid culture medium that only allows *ST* to grow. If bacterial colonies formed, it indicated that the model was established. Each mouse in the *L. de.* p + ST and *L. de.* i + ST groups was, respectively, administered with *L. de.* p and *L. de.* i at a dose of 5 × 10^8^ CFU every day. Additionally, each mouse in the *L. de.* s + ST group was orally administered with 200 μL of bacterial supernatant. During the experiment, we measured the body weights of the mice and collected feces, serum, and intestinal samples.

### 2.3. Histopathological Analysis

Fresh jejunum and colon were treated with 4% paraformaldehyde. After cleaning, the tissues were soaked in alcohol for dehydration. The intestine was placed in the center of a box containing paraffin and cooled to room temperature until the paraffin solidified. After embedding, paraffin sections were developed in water at 55 °C, and the slides were pasted and dried in an incubator at 55 °C for later use. The prepared paraffin sections were stained with hematoxylin and mounted with neutral gum. After drying, the intestinal morphology was observed using a virtual microscope.

### 2.4. Inflammatory Factors and Intestinal Permeability

Interleukin-10 (IL-10), Interleukin-6 (IL-6), tumor necrosis factor-α (TNF-α), D-Lactate (D-LA), diamine oxidase (DAO), and lipopolysaccharides (LPSs), as indicators of inflammatory factors and intestinal permeability, were detected by mouse source ELISA kits (Jiangsu Meimian Industrial Co., Ltd., Yancheng, China). All kits were performed according to the instructions.

### 2.5. RNA Extraction and Quantitative RT-PCR

RNA was extracted via the TRIzol method (Airlab Biotechnology Co., Ltd., Tokyo, Japan), diluted to the same concentration, and reverse transcribed (Novozan Biotechnology Co., Ltd., Nanjing, China) to obtain cDNA. Then, we determined the relative mRNA expression (Novozan Biotechnology Co., Ltd., Nanjing, China). The primers are listed in Supplementary Materials Table S1.

### 2.6. Western Blot

The proteins were extracted from mice jejunum and porcine intestinal organoids using RIPA lysis solution (Acmec) containing protease inhibitors (Alpha Diagnoestic International). Protein concentrations were determined using the BCA kit (Nanjing Jiancheng Biotechnology Co., Ltd., Nanjing, China) and then diluted to the appropriate concentrations. Protein samples were separated using electrophoresis and transferred to PVDF membranes (Abclonal). The membranes were blocked with 5% skimmed milk (SkimMilk) powder for 1 h and then incubated with primary antibodies and secondary antibodies (Abclonal). The water on the surface was absorbed using filter paper, incubated with the developer for 3 min, and photographed using a chemiluminescence imaging system.

### 2.7. Immunofluorescence Assay

The prepared paraffin sections were deparaffinized, treated with an antigen-repair solution, and permeabilized with 0.5% TritonX-100 (Beyotime). They were then incubated with the primary antibody (Servicebio) overnight after blocking with 5% BSA (Solarbio). The sample was then washed and incubated with the secondary antibody in the dark for 60 min. It was counterstained with DAPI (Servicebio) and left to stand before observation and acquisition by laser confocal scanning. ImageJ 1. X software was used to statistically process the images.

### 2.8. Intestinal Organoid Culture and Treatment

A 10 cm section of the jejunum was isolated from seven-day-old piglets. The intestinal segments were dissected longitudinally and washed repeatedly with PBS. The intestine was sliced into 5 mm sections and then treated with cell digestive enzymes (stem cell) at room temperature for 25 min. The supernatant was then discarded, 10 mL of PBS was added, and the mixture was shaken vigorously for 2 min. The cells were resuspended in complete medium (stem cell) and Matrigel (Coring) and then plated on 24-well plates. The cell status was recorded daily, and the medium was changed every three days. For the co-culture of *L. de.* i and organoids, after cell digestion, 1 × 10^4^ CFU of the inactivated bacterial precipitate was added to the Matrigel and plated. For the co-culture of bacterial supernatant and organoids, 10 μL of bacterial supernatant was added to the culture medium after plating.

### 2.9. 16S rRNA Sequencing

The total DNA was extracted from the cecal chyme samples and sent to Shanghai Meiji Biomedical Technology Co., Ltd. (Shanghai, China), for high-throughput 16S rRNA sequencing. The V3-V4 region of the bacteria 16S ribosomal RNA gene was amplified by using primers 341 F: 5′-ACTCCTACGGAGGGCAG-3′ and 806 R: 5′-GGACTACHVGGGTWTCTAAT-3′. PCR products were extracted by 2% agarose gel electrophoresis, purified using the AxyPrep DNA Gel Extraction Kit (Axygen Biosciences, Union City, CA, USA), and quantified using QuantiFluor™ Quantification System (Promega, Madison, WI, USA). The Illumina MiSeq was used for sequencing. The raw sequences were assembled and quality-controlled using FLASH software (https://ccb.jhu.edu/software/FLASH/index.shtml, version 1.2.11, accessed on 20 June 2023) and Fastp software (https://github.com/OpenGene/fastp, version 0.19.6, accessed on 20 June 2023). The sequences were clustered into OTU, and chimeras were removed based on 97% similarity, using UPARSE software (http://www.drive5.com/uparse/, version 11, accessed on 20 June 2023) and Unoise3 software (https://www.drive5.com/usearch/manual/unoise_algo.html/, version 11, accessed on 20 June 2023).

### 2.10. Determination of Short-Chain Fatty Acids (SCFAs)

After 24 h of infection with *ST*, fecal samples were pretreated with 25% *w*/*v* metaphosphoric acid, and the supernatant was collected after centrifugation at 12,000 rpm for 10 min. The gas chromatograph (Shimadzu, Tokyo, Japan) was used for detection after passing the supernatant through a 0.22 μm water system filter membrane.

### 2.11. Statistical Analysis

Statistical analysis was performed using SPSS 25.0 (SPSS Inc., Chicago, IL, USA), and graphs were plotted using GraphPad Prism 8.0 (La Jolla, CA, USA). One-way ANOVA and LSD were used for multiple comparison tests to assess statistical differences between 3 or more groups. Data were expressed as mean ± SEM. A *p* < 0.05 was considered statistically significant.

## 3. Results

### 3.1. Postbiotics from L. delbrueckii Prevented Body and Intestine Weight Loss in ST-Infected Mice

**Figure 1:**
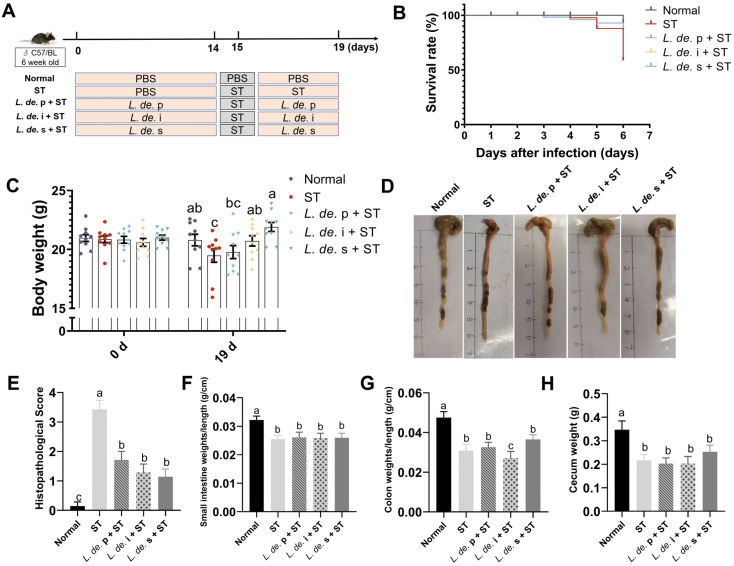
Effects of postbiotics from *L. delbrueckii* on the body weight and intestinal weight in *ST*-infected mice. (**A**) Experimental design. (**B**) Survival rate of mice treated with *ST*. (**C**) Body weight of mice on days 0 and 19. (**D**) Colonic morphology. (**E**) Histopathological score. (**F**) Small intestine weights per unit length. (**G**) Colon weights per unit length. (**H**) Cecum weight, *n* = 10. Data are presented as mean ± SEM; different letter superscripts indicate significant differences (*p* < 0.05).

To investigate whether the postbiotics from *L. delbrueckii* could prevent damage in *S*. Typhimurium-infected mice, we monitored the survival and weight changes in mice every two days. The results revealed that the mortality rate increased in mice infected with *ST*, whereas it decreased when treated with *L. de.* p, *L. de.* i, and *L. de.* s (Figure 1B). The weight of the mice decreased due to the *ST* challenge, and *L. de.* i and *L. de.* s increased the body weight of mice on the 19th day (*p* < 0.05, Figure 1C). Supplementation with *L. de.* p resulted in an increasing trend in mouse weight. Additionally, the weights of the small intestine, colon, and cecum were reduced in the ST group compared to those in the normal group, and postbiotics supplementation did not alleviate this symptom (Figure 1F–H).

### 3.2. Postbiotics from L. delbrueckii Improved Intestinal Morphology and Regulated Inflammation Factor Levels in ST-Infected Mice

**Figure 2:**
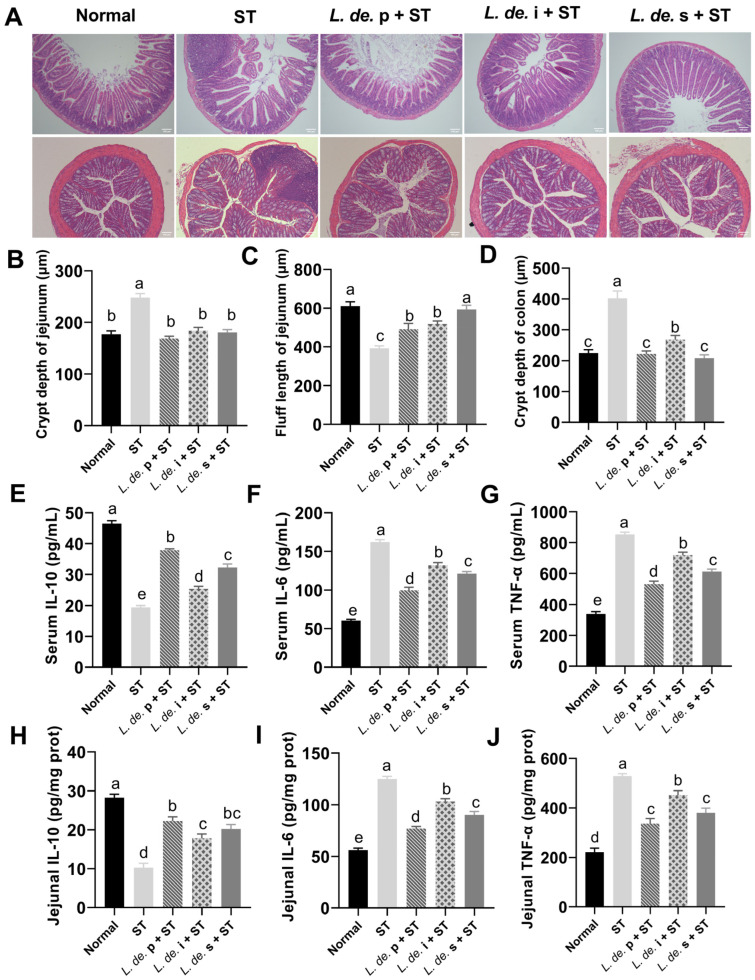
Effects of postbiotics from *L. delbrueckii* on intestinal morphology and inflammatory factors in *ST*-infected mice. (**A**) HE stain of the jejunum (upper **A**) and colon (lower **A**). Objective, ×10; scale bars = 100 μm. (**B**) Crypt depth of jejunum. (**C**) Fluff length of jejunum. (**D**) Crypt depth of colon. IL-10 (**E**), IL-6 (**F**), and TNF-α (**G**) of serum, IL-10 (**H**), IL-6 (**I**), and TNF-α (**J**) of jejunum, *n* = 7. Data are presented as mean ± SEM; different letter superscripts indicate significant differences (*p* < 0.05).

The intestinal morphology and inflammatory factor levels were measured. Compared to the normal group, the ST group showed elevated neutrophil infiltration, disrupted intestinal epithelial integrity, and decreased intestinal villi and microvilli (Figure 2A). Treatment with postbiotics alleviated the intestinal damage caused by *ST*, including increased jejunal fluff length and reduced jejunal and colonic crypt depths (*p* < 0.05, Figure 2B–D).

Compared to the normal group, the ST group decreased the IL-10 and increased TNF-α and IL-6 levels in serum (*p* < 0.05, Figure 2E–G). The *L. de.* p + ST, *L. de.* i + ST, and *L. de.* s + ST groups exhibited increased IL-10 and decreased TNF-α and IL-6 levels in serum compared to that of the ST group (*p* < 0.05, Figure 2E–G). Similar results were obtained by measuring the levels of inflammatory factor (Figure 2H–J).

### 3.3. Postbiotics from L. delbrueckii Modulated Intestinal Microbial Composition and Metabolites in ST-Infected Mice

**Figure 3:**
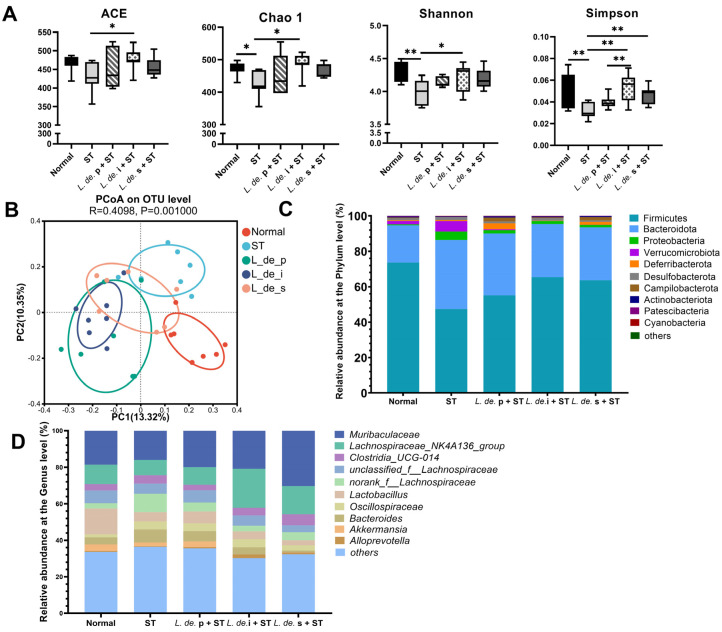
Effects of postbiotics from *L. delbrueckii* on intestinal microbial composition in *ST*-infected mice. (**A**) Alpha diversity index of cecal microbiota. (**B**) Principal coordinate analysis (PCoA) plot based on Bray−Curtis distance. (**C**) Stacked bar plots for the cecal microbiota at the phylum level. (**D**) Stacked bar plots for the cecal microbiota at the genus level, *n* = 7. Data are presented as mean ± SEM; * *p* < 0.05; ** *p* < 0.01.

To compare the gut microbial composition of mice in the different treatment groups, we selected cecal contents for 16S rRNA sequencing. The Chao, Shannon, and Simpson indices decreased in the ST group compared to those in the normal group; however, this trend was reversed after postbiotic treatment (*p* < 0.05, Figure 3A). The Beta diversity used by the principal coordinate analysis (PCoA) reflects the separation among different groups.

**Figure 4:**
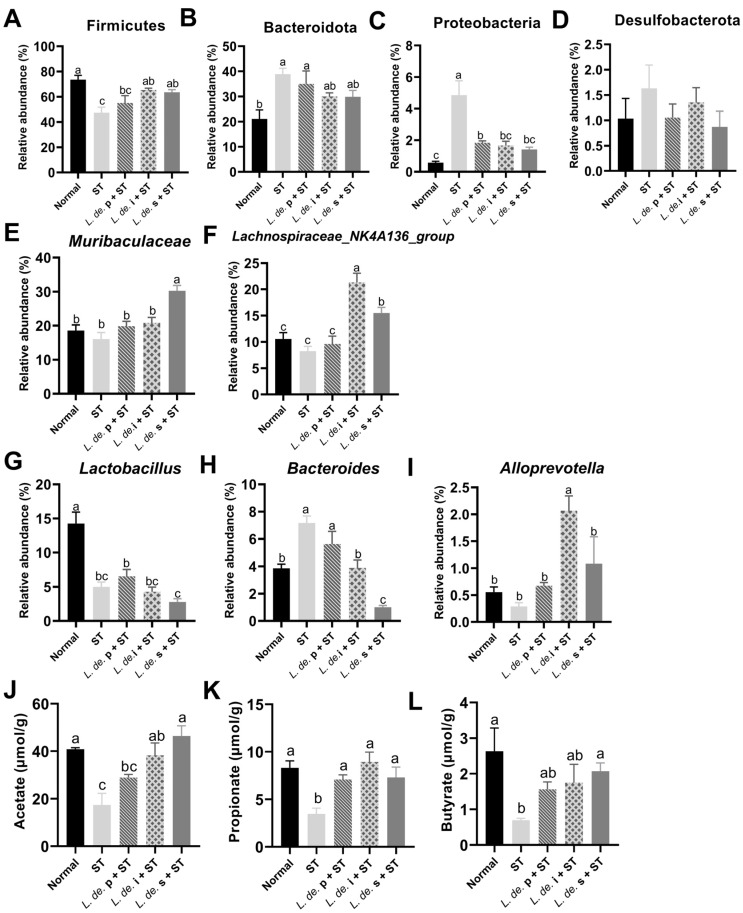
Effects of postbiotics from *L. delbrueckii* on differential bacteria and metabolites in *ST*-infected mice. Differential bacteria at the phylum (**A**–**D**) and the genus level (**E**–**I**). (**J**–**L**) Concentrations of acetate, propionate, and butyrate in feces after 24 h infection with *ST*, *n* = 7. Data are presented as mean ± SEM; different letter superscripts indicate significant differences (*p* < 0.05).

Among the collected samples, *Firmicutes* was the predominant phylum, followed by *Bacteroidetes* (Figure 3D). Compared to the normal group, the ST group showed a decrease in the abundance of *Firmicutes* (from 73.57% to 47.44%) with the addition of *L. de.* i and *L. de.* s, respectively (*p* < 0.05, Figure 4A). The *ST* treatment increased the abundance of *Bacteroides*, whereas treatment with *L. de.* p, *L. de.* i, and *L. de.* s decreased the abundance (Figure 4B). Additionally, we observed an enrichment of *Proteobacteria* in the ST group, which significantly decreased after exposure to *L. de.* p, *L. de.* i, and *L. de.* s (*p* < 0.05, Figure 4C). The abundance of *Desulfobacteria* exhibited the same trend as that of *Proteobacteria*.

According to the taxonomic profile at the genus level, *Muriaculaceae*, *Lachnospiraceae_NK4a136_group*, and *Lactobacillus* were the most abundant components in the cecal contents of mice (Figure 4C). Infection with *ST* reduced the relative abundance of *Muribacoccaceae*, *Lachnospiraceae_NK4a136_group*, *Lactobacillus*, and *Alloprevotella* and increased the abundance of Bacteroides (*p* < 0.05, Figure 4E–I). Compared to the ST group, the *L. de.* i + ST group exhibited an increased abundance of *Muribaculaceae* and *Alloprevotella* and reduced abundance of *Bacteroides* (*p* < 0.05, Figure 4E–I), and the *L. de.* s + ST group exhibited an increased content of *Lachnospiraceae_NK4a136_group* and a reduced abundance of Bacteroides (*p* < 0.05, Figure 4F–H). However, the *L. de.* p did not affect *Lactobacillus*.

The concentration of SCFA was detected in mice after 24 h of infection with *ST*. It was observed that the *ST* challenge resulted in a decrease in SCFA concentration. Compared to the ST group, the postbiotic groups possessed increased contents of acetate, propionate, and butyrate in the feces (*p* < 0.05, Figure 4J–L), and the *L. de.* p + ST group exhibited an increasing trend in acetate content.

### 3.4. Postbiotics from L. delbrueckii Enhanced Intestinal Barrier Function in ST-Infected Mice

**Figure 5:**
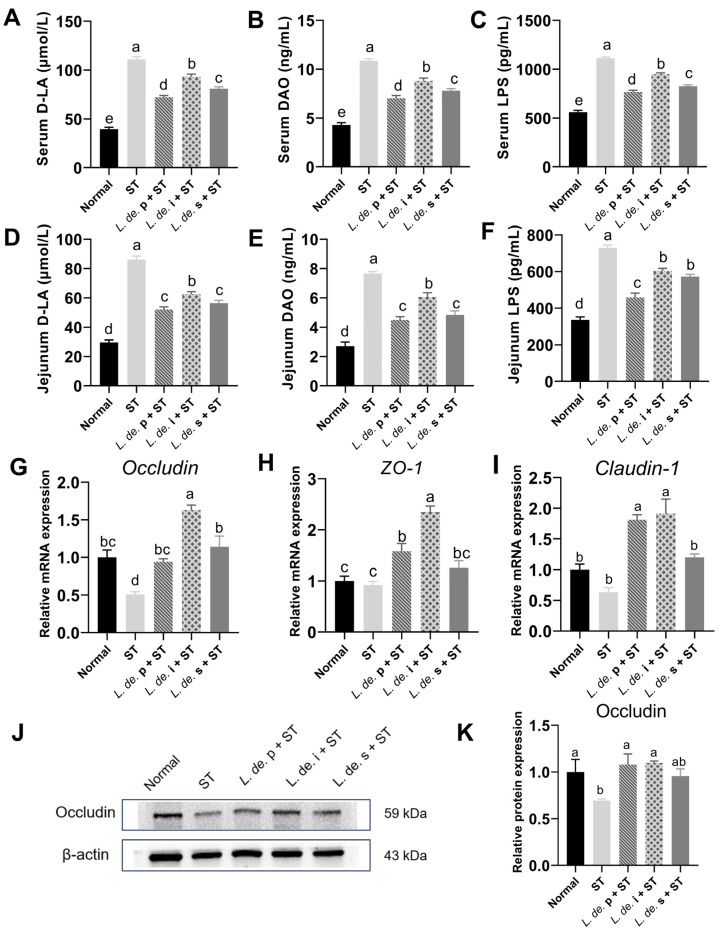
Effects of postbiotics from *L. delbrueckii* on intestinal barrier function in *ST*-infected mice: D-LA (**A**), DAO (**B**), and LPS (**C**) of serum; D-LA (**D**), DAO (**E**) and LPS (**F**) of jejunum. The mRNA expressions of *Occludin* (**G**), *ZO-1* (**H**), and *Claudin-1* (**I**), *n* = 6. (**J**) Protein expression of Occludin. (**K**) Relative protein expression of Occludin, *n* = 3. Data are presented as mean ± SEM; different letter superscripts indicate significant differences (*p* < 0.05).

The intestinal permeability and tight junction protein expression were measured to assess the effects of postbiotics on the intestinal barrier. The results indicated that the *ST* infection increased the levels of D-LA, DAO, and LPS in serum, and *L. de.* p and postbiotics treatment reduced the relevant indicators (*p* < 0.05, Figure 5A–C). Similar results in the jejunum were observed (Figure 5D–F).

*S*. Typhimurium treatment decreased the mRNA level of *Occludin* but did not affect the *ZO-1* and *Claudin-1* levels. Compared to the ST group, the *L. de.* p + ST and *L. de.* i + ST groups showed increased *Occludin*, *ZO-1*, and *Claudin-1* levels (*p* < 0.05, Figure 5G–I), and these indices were also increased in the *L. de.* s + ST group. The measurement of protein expression in the mice jejunum revealed that the expression of Occludin was decreased by *ST*, and postbiotic treatment increased Occludin expression (Figure 5J,K). These results suggested that postbiotics reduced intestinal permeability and improved the intestinal barrier.

### 3.5. Postbiotics from L. delbrueckii Promoted the Expansion of ISCs in ST-Infected Mice

**Figure 6:**
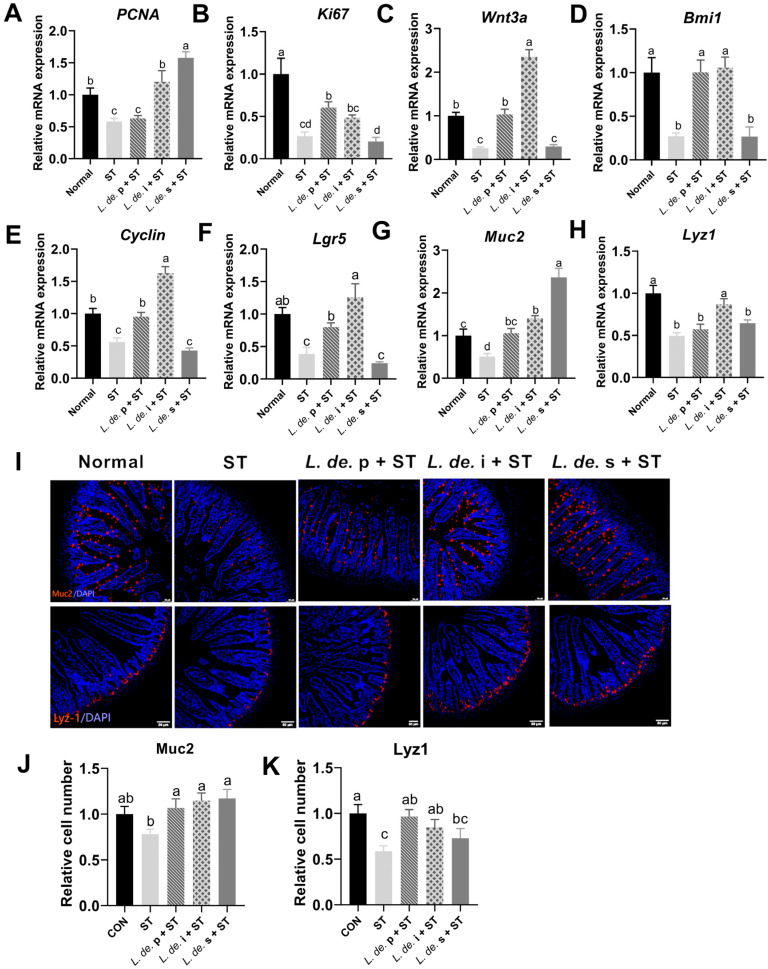
Effects of postbiotics from *L. delbrueckii* on intestinal stem cell expansion in *ST*-infected mice. The mRNA expressions of *PCNA* (**A**), *Ki67* (**B**), *Wnt3a* (**C**), *Bmi1* (**D**), *Cyclin* (**E**), *Lgr5* (**F**), *Muc2* (**G**), and *Lyz1* (**H**). (**I**) Immunofluorescence of Muc2 and Lyz1. Objective, ×20; scale bar, 50 μm. (**J**) Relative protein expression of Muc2. (**K**) Relative protein expression of Lyz1, *n* = 6. Data are presented as mean ± SEM; different letter superscripts indicate significant differences (*p* < 0.05).

Intestinal stem cells can self-renew and differentiate into different epithelial cells, and this may play a pivotal role in restoring damaged intestines. Infection with *ST* decreased the proliferation genes *PCNA*, *Ki67*, and *Cyclin*; the ISC marker gene *Lgr5*; the quiescent ISC marker gene *Bmi1*; the Goblet cell marker gene *Muc2*; the Paneth cell marker gene *Lyz1*; and *Wnt3a* mRNA levels (*p* < 0.05, Figure 6A–K). The *L. de.* p treatment increased the mRNA expression of *Ki67*, *Wnt3a*, *Bmi1*, *Cyclin*, and *Lgr5* and also increased the expression of *Muc2* and *Lyz1* (*p* < 0.05, Figure 6A–K). The *L. de.* i + ST group exhibited increased expression of *PCNA*, *Wnt3a*, *Bmi1*, *Cyclin*, *Lgr5*, *Muc2*, and *Lyz1* compared to that of the ST group (*p* < 0.05, Figure 6A–K). Additionally, the *L. de.* s + ST group exhibited increased *PCNA*, *Muc2*, and *Lyz1* expression, but there was no effect on *Ki67*, *Wnt3a*, *Bmi1*, *Cyclin*, and *Lgr5* compared to levels in the ST group (Figure 6A–K). We detected the marker protein expression of Goblet and Paneth cells in the jejunum by immunofluorescence and observed that *ST* ingestion reduced the expression of Muc2 and Lyz1, and the addition of *L. de.* p, *L. de.* i, and *L. de.* s relieved the inhibition of epithelial cells induced by the *ST* challenge. These results indicated that *L. de.* p and postbiotics promoted the expansion of ISCs and accelerated intestinal development.

### 3.6. Postbiotics from L. delbrueckii Accelerated the Proliferation and Differentiation of Porcine Intestinal Organoids

**Figure 7:**
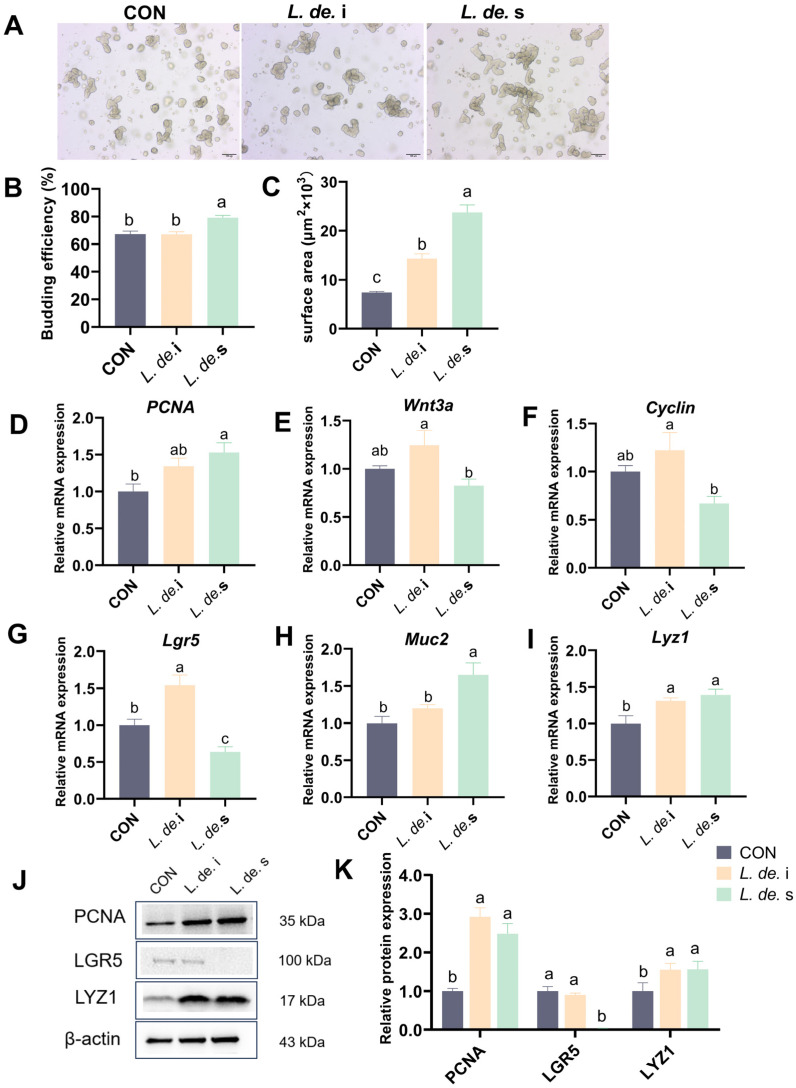
Effects of postbiotics from *L. delbrueckii* on proliferation and differentiation in porcine intestinal organoids. (**A**) Comparison of intestinal organoid morphology. Objective, ×4; scale bar, 100 μm. (**B**) Budding efficiency of organoids. (**C**) The surface area of organoids. The mRNA expressions of *PCNA* (**D**), *Wnt3a* (**E**), *Cyclin* (**F**), *Lgr5* (**G**), *Muc2* (**H**), and *Lyz1* (**I**), *n* = 4. (**J**) Protein expression. (**K**) Relative protein expression of PCNA, LGR5, and LYZ1, *n* = 3. Data are presented as mean ± SEM; different letter superscripts indicate significant differences (*p* < 0.05).

Small intestinal organoids from pigs were used as ex vivo models to verify the effects of postbiotics on the proliferation and differentiation of intestinal epithelial cells. The results revealed that, compared to the control, the *L. de.* i treatment showed an increase in the surface area of organoids, and the *L. de.* s treatment not only promoted the germination rate but also increased the surface area of organoids (*p* < 0.05, Figure 7A–C). Moreover, the *L. de.* i treatment promoted the mRNA expression of *PCNA*, *Wnt3a*, *Cyclin*, *Lgr5*, *Muc2,* and *Lyz1*, and *L. de.* s supplementation increased the expression of *PCNA*, *Muc2*, and *Lyz1* but tended to decrease *Cyclin*, *Wnt3a*, and *Lgr5* expression (Figure 7D,E). Detection of protein expression in the organoids revealed that the *L. de.* i and *L. de.* s groups exhibited enhanced expression of PCNA and LYZ1, and the *L. de.* i group did not affect LGR5. However, it was decreased in the *L. de.* s group. These results suggested that the treatment of postbiotics accelerated the proliferation and differentiation of intestinal organoids.

## 4. Discussion

*Salmonella* is a foodborne pathogen that causes intestinal inflammation in both humans and animals. Probiotics are a method for treating intestinal diseases, but they have some drawbacks, including uncertainty about the concentration of live bacteria, safety concerns, and unclear dosage requirements. As probiotic components, postbiotics exert a protective effect on the body [16,17,18]. In this study, intervention with probiotics and postbiotics from *L. delbrueckii* alleviated intestinal inflammation in *S.* Typhimurium-infected mice by promoting the proliferation and differentiation of the intestinal epithelium. Our results contribute to the treatment of human intestinal diseases using postbiotics.

As a type of probiotic, *Lactobacillus* is widely present in the gastrointestinal tract, and *L. delbrueckii* is one of these. Kanmani et al. reported that the probiotic effects of *L. delbrueckii* TUA4408L and its extracellular polysaccharides improve resistance against rotavirus infection by regulating the inflammatory response and reducing viral replication [19]. *L. delbrueckii* CIDCA 133 exhibited a protective effect in 5-fluorouracil-induced intestinal mucositis by reducing the loss of Goblet cells and inflammatory infiltration [20]. In the present study, *L. delbrueckii* increased mortality, significantly reduced intestinal pathology scores, increased intestinal villus height, decreased crypt depth, and improved intestinal histology in mice. The specific effects of *L. delbrueckii* lay the foundation for its use in the treatment of intestinal diseases.

Postbiotics are considered as alternatives to probiotics because of their stability and safety. They have been reported to enhance the intestinal barrier and modulate host immunity [21]. Li et al. observed that viable, inactivated, or lysed *L. plantarum* H6 reduced liver injury, modulated the microbiota composition, altered intestinal amino acid metabolism, decreased the relative concentration of primary bile acids, and effectively improved hypercholesterolemia in mice [22]. The postbiotics and probiotics of *Bifidobacterium adolescentis* have similar abilities to improve colitis induced by DSS, but they show different capacities to modulate intestinal microflora and metabolic pathways [23]. Additionally, the supernatant of *L. rhamnosus* GG upregulated the expression of serotonin transporters in the HT-29, Caco-2 cell line, and mice intestinal tissue, helping probiotics to alleviate IBS symptoms [24]. In this study, the inactivated *L. delbrueckii* and supernatants of *L. delbrueckii* improved the survival rate and decreased the weight loss of mice challenge by *S*. Typhimurium. The histopathological analysis revealed that *S*. Typhimurium caused infiltration of intestinal tissue cells and inflammation in mice. Postbiotic intervention alleviates intestinal damage and inflammation in mice. Therefore, probiotics alleviate the symptoms of intestinal inflammation caused by *Salmonella* through their components.

Pathogen invasion causes inflammatory cells to accumulate and release inflammatory factors, thereby promoting a series of inflammatory responses. *ST* challenge resulted in an increase in IL-6 and TNF-α in the serum and jejunum, which are key factors in inflammatory response and are widely involved in systemic inflammatory [25,26]. A decrease in IL-10 was also observed, which is essential for inhibiting excessive immune responses to pathogens, anti-inflammation, maintaining tissue epithelial integrity, and immune regulation [27]. Probiotics modulate the levels of inflammatory factors to protect against intestinal damage. *Bifidobacterium adolescentis* ameliorated DSS-induced colitis by reducing TNF-α, IL-1β, and IL-6 levels and increasing IL-10 and IL-4 levels [28]. *L. plantarum* decreased the levels of IL-1β, IL-6, TNF-α, and IFN-γ to enhance the immune barrier and effectively improve ulcerative colitis [29]. The present study confirmed that postbiotics from *L. delbrueckii* significantly decreased IL-6 and TNF-α levels and increased the IL-10 level in the serum and jejunum of mice. This indicated that postbiotics could affect the secretion of inflammatory factors.

The integrity of the intestinal barrier is crucial for protecting against damage caused by pathogens to the balance of the gut microbiota and intestinal mucosa. Several studies have demonstrated that postbiotics can help maintain intestinal barrier integrity. For instance, supplement of postbiotics from *L. plantarum* RG14 upregulates the mRNA expression of TJP-1, CLDN-4, and CLDN-1 to strengthen gut barrier integrity and gastrointestinal health in post-weaning lambs [30]. The postbiotics from *L. rhamnosus* enhance intestinal mucin expression and prevent intestinal damage induced by LPS in neonatal rats [31]. In this study, postbiotics from *L. delbrueckii* decreased the levels of D-LA, DAO, and LPS in the serum and jejunum and increased the mRNA expression of *Occludin*, *ZO-1*, and *Claudin-1* in jejunum, suggesting that postbiotics improved intestinal permeability and enhanced intestinal epithelial barrier function.

Unlike probiotics, postbiotics do not introduce new bacteria to the gut microbiota but often exhibit beneficial and therapeutic effects similar to those of probiotics [32]. This study observed that the decrease in the alpha-diversity index and the imbalance of gut microbiota due to *ST* challenge, inactivation, and the supernatant of *L. delbrueckii* increased gut microbial diversity and modulated the microbiota. *S*. Typhimurium invasion led to a decrease in the ratio of *Firmicutes* to *Bacteroidetes*, which indicated the development of intestinal inflammation [33]. Treatment with postbiotics from *L. delbrueckii* significantly increased the abundance of *Firmicutes* and decreased *Bacteroidetes*. At the genus level, postbiotics from *L. delbrueckii* restored the abundance of *Muribaculaceae*, *Lachnospiraceae_NK4a136_group*, and *Alloprevotella*. *Muribaculaceae* has been reported as a beneficial bacterium that can inhibit intestinal barrier dysfunction, intestinal inflammation, and lipid metabolism disorders in mice [34], and *Lachnospiraceae_NK4A136_group* produces SCFA through the fermentation of dietary polysaccharides to promote intestinal mucosal repair. This is negatively associated with several metabolic diseases and chronic inflammation in mice [35]. However, probiotics and postbiotics from *L. delbrueckii* did not affect *Lactobacillus*. Additionally, postbiotics decrease the abundance of *Bacteroides*, which can lead to intestinal flora disorders and intestinal inflammation [36]. The regulation of the microbiome may be an effective way to prevent and treat intestinal diseases [37]. It can be concluded that postbiotics from *L. delbrueckii* can modulate the diversity and abundance of flora and better promote human health.

Intestinal stem cells are situated at the base of intestinal crypts, playing a crucial role in shaping the structure and function of the intestinal epithelium [38]. ISCs suffer considerable damage due to intestinal inflammation, and Paneth and Goblet cells that secrete growth factors for ISCs may be affected [39]. In the present study, *S*. Typhimurium inhibited the expression of proliferation-related genes in ISCs, whereas *L. de.* p or *L. de.* i increased the expression of proliferation genes, including *PCNA*, *Ki67*, and *Cyclin*. Additionally, treatment with *L. de.* i upregulated the expression of *Lgr5* (ISC marker gene), *Bmi1* (ISC quiescent marker gene), and *Wnt3a* (ISC upstream marker gene), whereas *L. de.* s treatment inhibited those expressions. The specific active ingredients in the inactivated *L. delbrueckii* and its supernatants require further validation. Furthermore, supplementary *ST* reduces the number of Paneth and Goblet cells secreted by ISCs, thus limiting pathogen damage to the intestinal barrier [40]. We observed that *L. de.* i and *L. de.* s increased the mRNA and protein expression of Muc2 and Lyz1, which are markers of Goblet and Paneth cells [41,42]. These results suggest that postbiotics facilitate the expansion of ISCs.

To further validate the effects of postbiotics on intestinal epithelial function, we utilized an intestinal organoid model [43]. Previous studies have revealed that lactate produced by the microbiota significantly increases the expansion of ISCs and the proliferation of Paneth and Goblet cells [44]. A recent study reported that only supplementation with live *L. reuteri* effectively activated the proliferation of intestinal epithelial cells and protected the morphology of organoids [45,46]. Further exploration revealed that postbiotics from *L. delbrueckii* tended to increase the budding efficiency and surface area of organoids and upregulated the expression of Muc2 and Lyz1, implying that postbiotics not only promoted the proliferation of intestinal organoids but also accelerated their differentiation.

Although the role of postbiotics has been well understood in recent years, there has been little research on human intestinal diseases. Our research introduces a novel concept of using postbiotics as a potential treatment for intestinal diseases. However, various factors may contribute to limitations in the research. Since the preparation method of postbiotics has been improved based on previous studies, it may impact the composition and content of postbiotics. In addition, we did not identify effective ingredients in the inactivated *L. delbrueckii* and its supernatant, which may help to explain the mechanism of postbiotics.

## 5. Conclusions

In conclusion, this study demonstrated that postbiotics from *L. delbrueckii* alleviated the *S*. Typhimurium-induced intestinal inflammatory response by promoting the proliferation and differentiation of ISCs. These findings contribute to our understanding of the potential use of postbiotic supplementation in improving intestinal health.

## Data Availability

The original contributions presented in the study are included in the article/Supplementary Materials, further inquiries can be directed to the corresponding author.

## Supplementary Materials

The following supporting information can be downloaded at https://www.mdpi.com/article/10.3390/foods13060874/s1, Table S1: List of primers used in the present study.
