# Supplementary material for: Postbiotics from Lactobacillus delbrueckii Alleviate Intestinal Inflammation by Promoting the Expansion of Intestinal Stem Cells in S. Typhimurium-Induced Mice
Source: Foods. 2024 Mar 14;13(6):874. doi: 10.3390/foods13060874 (PMC10969446; doi:10.3390/foods13060874)
Supplement: Supplementary file 1 [file foods-13-00874-s001.zip › foods-2909719-supplementary.pdf]

Supplementary table S1

Table S1. List of primers used in the present study

Primers	Forward primer (5'-3')	Reverse primer (5'-3')
m-GAPDH	F: AGGCCGGTGCTGAGTATGTC	R: GGCGGAGATGATGACCCTT
m-Occludin	F: ATCGTGGCTTTTGCTTTAATCATC	R: GGGCTGTTCATCATAAATGTGTT
m-ZO-1	F: ACCGAAACCTGTGTATGCTC	R: ATCATTTCCACCAGCTAGTCG
m-Claudin-1	F: TGGGAGGTGTCCTACTTTCCT	R: TTCCGATAACCATCATCAACAG
m-PCNA	F: TGCTCTGAGGTACCTGAACT	R: TGCTTCCTCATCTTCAATCT
m-Ki67	F: ACCGTGGAGTAGTTTATCTG	R: TGTTTCCAGTCCGCTTACTT
m-Cyclin	F: CATGTATCATCTAGCCATGCA	R: ATGCACAACAGGCCGCTAC
m-Lgr5	F: CCTTCACAGCCTCAAAGTG	R: GCAGGGATTGAAGGCTTCT
m-Muc2	F: ACGATGCCTACACCAAGGTC	R: TGATCTTCTGCATGTTCCCA
m-Lyz1	F: AGGAATGGAATGGATGGCTA	R: CGGTCTCCACGGTTGTATT
m-Wnt3a	F: CTCGCTGGCTACCCAATTTG	R: CTTCACACCTTCTGCTACG
m-Bmi1	F: TTCATTGTCTTTTCCGCCCG	R: AGTACCCTCCACACAGGAC
p-GAPDH	F: CCTATAGCCGTCCATGCCAG	R: CCTATAGCCGTCCATGCCAG
p-PCNA	F: TACGCTAAGGGCAGAAGATAATG	R: CTGAGATCTCGGCATATACGTG
p-Cyclin	F: GCCCTCCGTGTCCTACTTCA	R: AGACCTCCTCCTCGCACTTCT
p-Lgr5	F: GCCTTTGTAGGCAACCCTTC	R: AGGCACCATTCAAAGTCAGTG
p-Muc2	F: AGACGGGCGGAGACTTTGAATC	R: CTTGGATGGGAACGCTGGGATA
p-Lyz1	F: CCCGGCTTCTCAGACAACAT	R: CCTATAGCCGTCCATGCCAG
p-Wnt3a	F: GAGTGCCAACACCAGTTCC	R: AGTCACAGCGAAGGCAACTC
